# Bidirectional de novo peptide sequencing using a transformer model

**DOI:** 10.1371/journal.pcbi.1011892

**Published:** 2024-02-28

**Authors:** Sangjeong Lee, Hyunwoo Kim

**Affiliations:** Center for Biomedical Computing, Korea Institute of Science and Technology Information, Daejeon, Republic of Korea; University of Antwerp, BELGIUM

## Abstract

In proteomics, a crucial aspect is to identify peptide sequences. De novo sequencing methods have been widely employed to identify peptide sequences, and numerous tools have been proposed over the past two decades. Recently, deep learning approaches have been introduced for de novo sequencing. Previous methods focused on encoding tandem mass spectra and predicting peptide sequences from the first amino acid onwards. However, when predicting peptides using tandem mass spectra, the peptide sequence can be predicted not only from the first amino acid but also from the last amino acid due to the coexistence of *b*-ion (or *a*- or *c*-ion) and *y*-ion (or *x*- or *z*-ion) fragments in the tandem mass spectra. Therefore, it is essential to predict peptide sequences bidirectionally. Our approach, called NovoB, utilizes a Transformer model to predict peptide sequences bidirectionally, starting with both the first and last amino acids. In comparison to Casanovo, our method achieved an improvement of the average peptide-level accuracy rate of approximately 9.8% across all species.

## Author summary

Understanding the characteristics of data is very important in deep learning methods. When predicting sentences, the transformer model naturally predicts from the first word. For this reason, previous methods predicted peptide sequences from the first amino acid. However, in tandem mass spectra, it is possible to predict peptide sequences bidirectionally. This method shows better results than previous approaches because it can better encode tandem mass spectra. We have demonstrated that good results can be achieved simply by understanding the characteristics of such data and using the model appropriately. We hope that this paper will help various readers improve the performance capabilities of their models. Furthermore, given that bidirectional peptide sequence prediction is crucial in de novo peptide sequence analysis, we hope that this approach will be applied to both existing and future methods utilizing deep learning techniques.

## Introduction

In proteomics, the identification of peptide sequences is of paramount importance. To identify peptide sequences, proteins are initially digested to peptide fragments. Subsequently, tandem mass spectra are generated by the digested peptides [1]. The generated tandem mass spectra are then utilized for peptide sequence identification.

The two most widely used methods for identifying peptide sequences in proteomics are de novo sequencing and a database search [2–7]. De novo sequencing relies on the unique characteristics of tandem mass spectra to determine peptide sequences. On the other hand, the database search method involves searching for the most similar peptide sequences in a database using the tandem mass spectra to identify peptide sequences. The database search method is fast and accurate when used to identify peptide sequences that already exist in a database, but it is limited in its ability to identify peptide sequences that are not present in a protein database. Therefore, de novo sequencing becomes essential when identifying peptide sequences that are not in a protein database or when dealing with peptide sequences for which a suitable protein database is unavailable.

Over the past two decades, several tools, such as PepNovo [8], PEAKS [3], and Novor [9], have been proposed for de novo sequencing. More recently, there has been growing interest in utilizing deep learning techniques for de novo sequencing. DeepNovo [10] introduced a method that combines a convolutional neural network (CNN) and a recurrent neural network (RNN) [11,12] achieving superior results compared to methods that do not use deep learning across various species. Building on the progress made by DeepNovo, the same research team proposed PointNovo [13], which offers a faster and more accurate approach for high-resolution tandem mass spectra compared to DeepNovo. Furthermore, Casanovo [14] and InstaNovo [15], which use a Transformer model [16] of the type generally used for natural language processing, were also proposed^13^. Recently, BiATNovo [17] and GraphNovo [18] were suggested. BiATNovo employ a combination of a CNN and an attention function, and GraphNovo uses a combination of a graph neural network (GNN) [19] and an attention function. BiATNovo utilizes a bidirectional peptide sequencing method as a way to solve the output unbalance and deviation accumulation phenomenon caused by one-way predictions in existing de novo sequencing methods. The GraphNovo model was proposed to address the missing fragmentation problem by means of a two-step analysis. This model uses graph neural networks to not only resolve the missing fragmentation problem but also to predict peptides effectively.

Most previous methods focused on the encoding of tandem mass spectra and predictions of peptide sequences from the first amino acid onwards. However, when predicting peptides using tandem mass spectra, the peptide sequence can be predicted not only from the first amino acid but also from the last amino acid owing to the coexistence of *b*-ion (or *a*- or *c*-ion) and *y*-ion (or *x*- or *z*-ion) fragments in tandem mass spectra. Therefore, it is essential to predict peptide sequences bidirectionally. DeepNovo uses two separate sets (forward and backward) of parameters for bidirectional sequencing. BiATNovo employs bidirectional synchronous prediction, inspired by the bidirectional synchronous generation of natural language [20,21], for efficient predictions of the amino acid at a specific position in the middle using the forward and backward prediction sequences.

Our method is similar to existing methods that use a Transformer model, but it predicts the peptide sequence more accurately than these methods by modifying a total of three parts. First, for the encoder, the precursor mass, charge, *m/z*, and intensity were used as input values. Second, for the decoder, the residual mass is additionally concatenated and used while predicting amino acids. Third, the key feature of our approach is the bidirectional prediction of peptide sequences, which is achieved using two decoders. This means that we predict peptide sequences from both the first and last amino acid using two decoders in the single model. Our method demonstrated an average peptide-level accuracy improvement of approximately 9.8% over Casanovo across all species. Moreover, it outperformed DeepNovo by 17.4% and PointNovo by 11.1% in terms of the peptide-level accuracy.

## Datasets and evaluation metric

We used nine datasets acquired from Thermo Scientific Q-Exactive with HCD peptide fragmentation [22–30]. These datasets were first used in the DeepNovo paper and then used equally in PointNovo and Casanovo. The species tested were Mouse, Human, Yeast, M. mazei, Honeybee, Tomato, Rice bean, Bacillus, and Clam bacteria (Table A in S1 Text). For these datasets, the PEAKS DB was run, with fixed modification of the carbamidomethylation of cysteine (C) and variable modifications of the oxidation of methionine (M) and deamidation of asparagine (N) and glutamine (Q).

**Fig 1:**
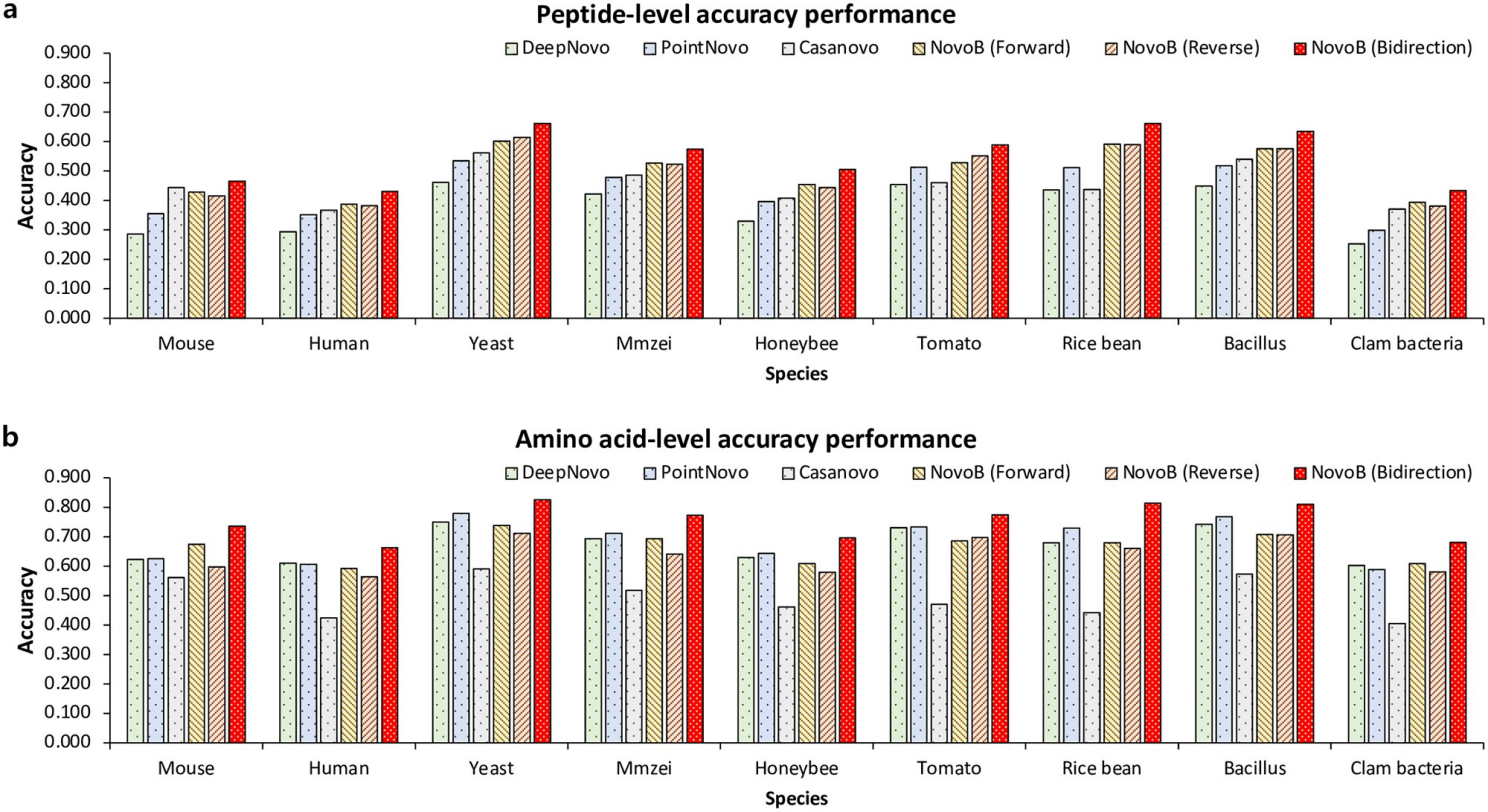
Comparison of DeepNovo, PointNovo, Casanovo, NovoB (Forward), NovoB (Reverse) and NovoB (Bidirection). These figures show the peptide-level and amino acid-level accuracy rates of six models on all nine species.

We used data from the nine species (Mouse, Human, Yeast, M. mazei, Honeybee, Tomato, Rice bean, Bacillus, Clam bacteria) in Table A in S1 Text and used the existing leave-one-out cross-validation method for comparison. This method uses eight species to learn and one species to verify. We compared the amino acid and peptide-level accuracy rates of the proposed method with DeepNovo, PointNovo, and Casanovo (Fig 1 and Table C in S1 Text). We used the same evaluation metric adopted by PointNovo and Casanovo when calculating the amino acid and peptide-level accuracy rates. The amino acid-level accuracy is *N*_*match*_*/N*_*predict*_, where *N*_*match*_ is the number of predicted amino acids matching actual amino acids and *N*_*predict*_ is the number of predicted amino acids. A predicted amino acid matches a real amino acid if their mass difference is smaller than 0.1Da and if the prefix masses before them are different by less than 0.5Da. The peptide-level accuracy is *N*_*match*_*/N*_*predict*_, where *N*_*match*_ is the number of predicted peptides matching actual peptides and *N*_*predict*_ is the number of predicted peptides.

## Result

### Bidirectional prediction

A tandem mass spectrum may contain both types (*e*.*g*., *b*-ion and *y*-ion) of fragments, and sometimes only one type of ion, but the methods proposed thus far only predict peptide sequences in one direction. NovoB selects a peptide sequence with a higher probability by predicting both forward and reverse peptide sequences using two decoders. As shown in Fig 1, we compared the accuracy of the prediction results in the three cases (when only the forward peptide sequence is predicted, when only the reverse peptide sequence is predicted, and when a peptide sequence with a high probability is selected between the forward and reverse peptide sequences).

Compared to other models, the forward peptide-level accuracy of NovoB increased by approximately 12.2% (DeepNovo), 5.9% (PointNovo), and 4.6% (Casanovo) on average. The reverse peptide-level accuracy of NovoB increased by approximately 12.1 (DeepNovo), 5.8% (PointNovo), 4.4% (Casanovo) on average. The peptide-level accuracy of NovoB is improved on average compared to the three models, regardless of the forward and reverse peptide-level accuracy outcomes. Furthermore, when compared to the other models, the forward amino acid-level accuracy of NovoB decreased by approximately 0.8% for DeepNovo and 2.2% for PointNovo and increased by approximately 17.1% (Casanovo) on average. The reverse amino acid-level accuracy of NovoB decreased on average by approximately 3.6% for DeepNovo and 5.0% for PointNovo, whereas it increased on average by approximately 14.3% for Casanovo.

For forward and reverse peptide levels, NovoB show higher accuracy for all species compared to DeepNovo and PointNovo, but compared to Casanovo, these peptide accuracy rates only decreased by about 1.5% (forward) and 2.8% (reverse) for mouse. Furthermore, for the forward and reverse amino acid levels, NovoB show higher accuracy for all species than Casanovo. For the forward amino acid level, the accuracy of NovoB compared to DeepNovo decreased for six species (human, yeast, M mazei, honeybee, tomato, and bacillus), and compared to PointNovo, it decreased for seven species (human, yeast, M mazei, honeybee, tomato, rice bean, and bacillus). For the reverse amino acid level, the accuracy of NovoB compared to DeepNovo and PointNovo decreased for all species.

However, the bidirectional peptide-level accuracy of NovoB increased by approximately 17.4% (DeepNovo), 11.1% (PointNovo), and 9.8% (Casanovo) on average, and the bidirectional amino acid-level accuracy increased by approximately 7.9% (DeepNovo), 6.5% (PointNovo), and 25.8% (Casanovo) on average. Casanovo has cases in which the accuracy decreases compared to the peptide-level accuracy of PointNovo for two species (tomato and rice bean), while the accuracy decreases in all cases of amino acid-level accuracy. However, compared to DeepNovo, PointNovo, and Casanovo, NovoB as proposed here shows higher accuracy for both peptide and amino acid-levels across all species.

We additionally used GraphNovo’s dataset to compare the peptide and amino acid-level accuracy rates of DeepNovo, PointNovo, Casanovo, GraphNovo and NovoB. GraphNovo used the HeLa and Cerebellum datasets for training, the Plasma dataset for validation, and the A. thaliana, C. elegans, and E. coli datasets for testing [31–34] (for details, see Table B and Fig B in S1 Text). The bidirectional peptide-level accuracy of NovoB increased by approximately 20.4% (DeepNovo), 8.0% (PointNovo), and 13.3% (Casanovo) on average. When compared to GraphNovo, NovoB showed an increase in the peptide-level accuracy for E. coli, though it showed a decrease for A. thaliana and C. elegans. Additionally, the bidirectional amino acid-level accuracy rates of NovoB increased by approximately 7.6% (DeepNovo), 1.3% (PointNovo), and 22.4% (Casanovo) on average. In comparison to GraphNovo, NovoB presents lower accuracy rates across three species.

### Residual mass

**Fig 2:**
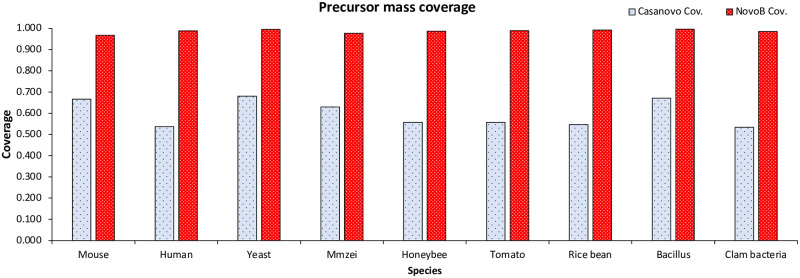
Comparison of coverage outcomes between Casanovo and NovoB. These figures are the precursor mass coverage for all nine species.

For the predicted peptide to be the actual correct answer, it must have the precursor mass as the tandem mass spectra within the tolerance limit. However, for Casanovo, as shown in Fig 2, only about 59.8% of the total results predicted the precursor mass within the tolerance level, while the remaining 39.8% failed to predict the precursor mass within tolerance. Compared to the database search, which selects only peptides corresponding to the precursor mass within the tolerance level, Casanovo has a high rate of not predicting the precursor mass within tolerance. To solve this problem, various methods use the knapsack algorithm effectively to address precursor-mass mismatches by ensuring that all sequences match the precursor mass. However, we conducted training while including the residual mass in the decoder input. Consequently, as shown in Fig 2, approximately 98.6% of the total results predicted the precursor masses within tolerance. That is, only about 1.4% failed to predict the precursor mass within tolerance.

**Fig 3:**
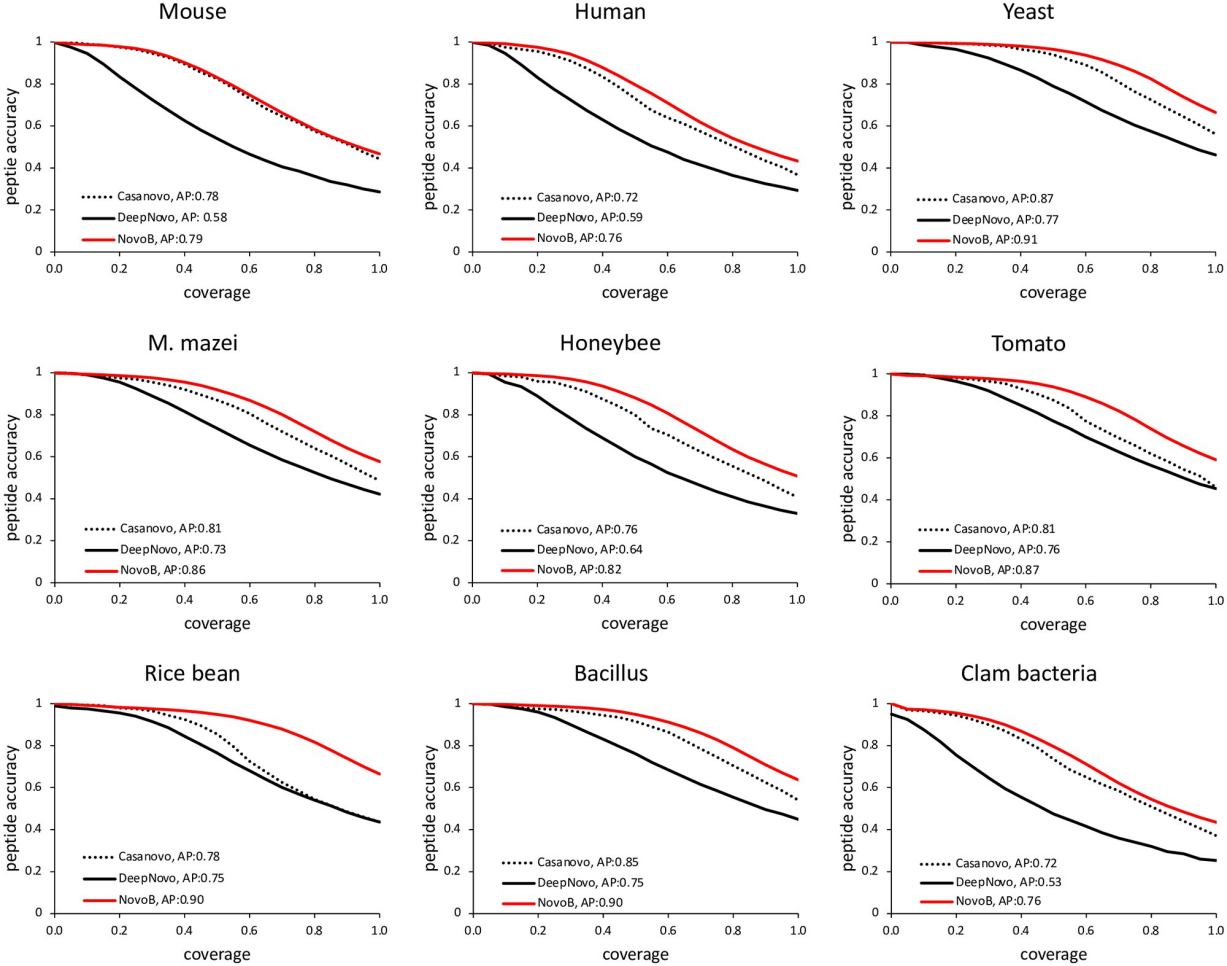
Accuracy-coverage curve for DeepNovo, Casanovo and NovoB. This figure evaluates peptide-level accuracy rates across all coverages for nine species. The x-axis represents the peptide-level accuracy, while the y-axis denotes the coverage. Additionally, the AUC value is expressed for each curve.

To evaluate the accuracy against DeepNovo and Casanovo at the same coverage level, we measured the accuracy-coverage curve and AP (average precision) by applying the coverage of each species to NovoB to compare this outcome. Consequently, compared to DeepNovo and Casanovo, our approach showed an increase in the AP values (Fig 3).

### Ablation study

We explored the factors contributing to the enhanced performance of the proposed method through an ablation study of NovoB (Table 1). We conducted a comparison between scenarios where the residual mass was included as the decoder input and when it was omitted. This comparison involved cases where only the forward decoder was utilized, where only the reverse decoder was utilized, and where both the forward and reverse decoders were utilized simultaneously. We also used the leave-one-out cross-validation method for this comparison, where we used yeast data to verify and eight species to learn.

**Table 1:** Comparison between Scenarios with the Inclusion and Exclusion of the Residual Mass as the Decoder Input.

	Peptide-level accuracy			Amino acid-level accuracy		
	NovoB (Forward)	NovoB (Reverse)	NovoB (Bidirection)	NovoB (Forward)	NovoB (Reverse)	NovoB (Bidirection)
**Not including** **residual mass**	0.590	0.589	0.627	0.685	0.668	0.712
**Including** **residual mass**	0.601	0.613	0.661	0.739	0.712	0.825

For peptide-level accuracy, when including the residual mass as the decoder, the input increased by approximately 1.1% (Forward), 2.4% (Reverse), and 3.4% (Bidirection). For the amino acid-level accuracy, when including the residual mass as the decoder, the input increased by approximately 5.4% (Forward), 4.4% (Reverse), and 11.3% (Bidirection). Regarding the peptide and amino acid-level accuracy rates, when the decoder includes the residual mass, it shows higher accuracy than when it does not.

In particular, when the residual mass was used as the decoder input, approximately 99.5% of the results overall predicted the precursor mass within the tolerance level. In contrast, when the residual mass was not used as the decoder input, only about 76.1% of the results in total predicted the precursor mass within the tolerance level.

### Conclusion and discussion

Our method, NovoB, utilizes the Transformer model to predict peptide sequences bidirectionally. The tandem mass spectrum may contain both *b*-ion (or *a*- or *c*-ion) and *y*-ion (or *x*- or *z*-ion) fragments, and sometimes only one type of ion. By predicting peptide sequences bidirectionally, our approach proves to be more effective when used to identify peptide sequences compared to conventional methods. Across all species, our method demonstrates an average peptide-level accuracy improvement of approximately 9.8% over Casanovo.

## Methods

Our method is similar to existing methods that use a Transformer model, but it predicts the peptide sequence more accurately than these methods by modifying a total of three parts. First, for the encoder, the precursor mass, charge, *m/z*, and intensity were used as input values. The precursor mass and m/z were encoded by means of positional encoding. Regarding the charge, it was also encoded with positional encoding. For intensity, we used only the integer part by multiplying it by 100 after normalization using the base peak. We concatenated the obtained precursor mass and charge for use as a single vector (only one). Also, the obtained *m/z* and intensity outcomes were concatenated for use as a single vector (according to the number of peaks). Second, for the decoder, the residual mass is additionally concatenated and used while predicting amino acids. At this time, the residual mass was also encoded using positional encoding. Third, the key feature of our approach is its ability to undertake the bidirectional prediction of peptide sequences, which is achieved using two decoders. One decoder predicts the forward peptide sequence, while the other predicts the reverse peptide sequence. Unlike traditional Transformer models, which typically predict only forward text as language models, it is crucial in de novo peptide sequencing to train the model to predict both forward and reverse sequences. This is essential because tandem mass spectra may contain both *b*-ion (or *a*- or *c*-ion) and *y*-ion (or *x*- or *z*-ion) fragments, or sometimes only one type of ion.

With bidirectional peptide sequence predictions, our method can more effectively identify peptide sequences compared to conventional approaches. After employing two decoders for the bidirectional peptide sequence prediction, we select the peptide sequence with the higher probability as the final prediction.

### Model architecture

Our method uses the Transformer model. Fig A in S1 Text shows the model of NovoB. The NovoB model consists of one encoder and two decoders in the Transformer model. The inputs of the encoder are the precursor mass, charge, and tandem mass spectrum (*m/z* and intensity). The inputs of the decoder are the amino acid sequence and the residual mass. The forward decoder predicts the forward peptide sequence, and the reverse decoder predicts the reverse sequence. Ultimately, the peptide sequence with a higher probability between those predicted by the forward and reverse decoders is selected.

### Encoding

Our method mostly encodes various values. We use positional encoding as used in the Transformer model for encoding. This is done as shown below.


$$E_{\left(k,2i\right)}=\text{sin}\left(k/{{Max}_{k}}^{\frac{2i}{d}}\right)$$

$$E_{\left(k,2i+1\right)}=\text{cos}\left(k/{{Max}_{k}}^{\frac{2i}{d}}\right)$$

Here, *k* denotes the values of the precursor mass, charge, *m/z*, and intensity; *i* is the dimension; *Max*_*k*_ is the max of *k*; and *d* is the size of the encoding vector.

### Encoder input

The encoder input uses the vector obtained after *Start Encoding* and *Peak Encoding*.

### *Start Encoding* of the encoder

*Start Encoding* is used by concatenating the vectors obtained from *precursor mass encoding* and *charge encoding*.

### Precursor mass encoding

We calculated *PM*(*p*) for encoding as follows:

PM(p)=(p×resolution)

In these equations, *p* is the precursor mass and *resolution* is the resolution of the tandem mass spectra. Considering the tolerance, we used 1,000 for the value of *resolution* to represent up to three decimal places. *PM*(*p*) was encoded by means of encoding.

### Charge encoding

*Charge encoding* encodes the charge by means of encoding.

### *Peak encoding* of the encoder

*Peak encoding* concatenates the vectors obtained from *m/z encoding* and *intensity encoding*.

### m/z encoding

We calculated MZ(*m*/*z*) for encoding as follows:

MZ(m/z)=(m/z×resolution)

Here, *m/z* is the *m/z* value and *resolution* is the resolution of the tandem mass spectra. Considering the tolerance, we used 1,000 for the value of *resolution* to represent up to three decimal places. MZ(*m*/*z*) was encoded by means of Encoding.

### Intensity encoding

Our method also encodes the intensity values. *Int*_(*I*)_ was calculated as follows:

$${SI}_{\left(I\right)}=sqrt\left(I\right)$$

$${Int}_{\left(I\right)}=floor\left({SI}_{\left(I\right)}/basepeak\times 100\right)$$

In these equations, *I* is the value of the intensity, *sqrt* is the function that calculates the square root, *floor* is the function which truncates the values into integers, and *basepeak* is the max of *SI*(*I*). *Int*_(*I*)_ was encoded by means of Encoding.

### Decoder input

The decoder input is used by amino-acid embedding and *residual mass encoding*.

### Residual mass encoding

We calculated *RM*(*r*) for encoding as follows:

RM(r)=(r×resolution)

Here, *r* is the residual mass and *resolution* is the resolution of the tandem mass spectra. Considering the tolerance, we used 1,000 for the value of *resolution* to represent up to three decimal places. *RM*(*r*) was encoded by means of Encoding.

### Hyper parameter

We train models with a total of ten encoder and decoder layers. The total size (*d*) of the input vector of the Encoder and Decoder is 64. There are two main types of input vector for the Encoder. The vector of *start encoding* includes information about the precursor mass and charge, and the vector of *peak encoding* includes information about the *m/z* and intensity. At this time, the size of the *start encoding* vector is 64, which is the concatenation value of the sizes of the *precursor mass encoding* vector (60) and the *charge encoding* vector (4). The max values of the precursor mass and charge are 8,000 and 20, respectively. In addition, the size of the *peak encoding vector* is 64, which is the concatenation value of the sizes of the *m/z encoding* vector (60) and the *intensity encoding* vector (4). The max values of *m/z* and intensity are 8,000 and 150, respectively. These vectors (*start encoding* vector (only one) and *peak encoding* vector (according to the number of peaks)) are utilized as the input vector for the Encoder. The *start encoding* vector (64) = precursor mass (60) + charge (4), the *peak encoding* vector (64) = m/z (60) + intensity (4). Also, the input vector of the Decoder includes information about the amino acid and residual mass. The size of Decoder input vector is 64, which is the concatenation value of the size of the *residual mass encoding* vector (60) and the *amino acid embedding* vector (4). The max value of residual mass is 8,000. The input vector of the Decoder (64) = amino acid (4) + residual mass (60). The size of the multi-head vectors is 64, and there are eight heads, yielding a total of ~12M model parameters. NovoB has significantly fewer parameters compared to Casanovo at 47M and DeepNovo at 86M, with the total number of parameters being 12M (not reported for PointNovo). We used a batch size of 256. We used the formula below to vary the learning rate during the training process. This method increases the learning rate linearly for the first warmup_steps training step and decreases the learning rate proportional to the inverse square root of the number of subsequent steps. Here, warmup_steps = 32K.


$$learning\;rate=v^{-0.5}\times min({sn}^{-0.5},sn\times {ws}^{-1.5})$$

In this equation, *v* is the vector size, *sn* is the number of steps, and *ws* denote warmup_steps. The model was trained for approximately one day for 30 epochs on a single NVIDIA A100 GPU, and the model hyperparameters were applied identically in all experiments (Table D in S1 Text).

## Supporting information

S1 Text NovoB supplementary information. (DOCX)
