# Supplementary material for: Bidirectional de novo peptide sequencing using a transformer model
Source: PLoS Comput Biol. 2024 Feb 28;20(2):e1011892. doi: 10.1371/journal.pcbi.1011892 (PMC10901305; doi:10.1371/journal.pcbi.1011892)
Supplement: S1 Text — (DOCX) [file pcbi.1011892.s001.docx]

**Table A in S1 Text |** Summary of nine datasets used by Casanovo.

|  | No. of spectra | Precursor mass  tolerance (PPM) | Accession no. |
| --- | --- | --- | --- |
| Mouse | 36,920 | 10 | PXD004948 |
| Human | 123,137 | 20 | PXD004424 |
| Yeast | 100,114 | 20 | PXD003868 |
| M. mazei | 161,593 | 10 | PXD004325 |
| Honeybee | 304,710 | 20 | PXD004467 |
| Tomato | 279,096 | 15 | PXD004947 |
| Rice bean | 36,186 | 20 | PXD005025 |
| Bacillus | 278,033 | 30 | PXD004565 |
| Clam bacteria | 138,085 | 20 | PXD004536 |

**Table B in S1 Text** | Summary of three datasets used by GraphNovo.

|  | No. of spectra | Precursor mass  tolerance (PPM) | Accession no. |
| --- | --- | --- | --- |
| HeLa | 410,366 | 5 | PXD006109 |
| Cerebellum | 1,249,397 | 5 | PXD006109 |
| Plasma | 20,472 | 5 | PXD006109 |
| A. thaliana | 12,222 | 5 | PXD016315 |
| C. elegans | 12,103 | 5 | PXD013233 |
| E. coli | 12,330 | 5 | PXD016001 |

**Additional datasets**

We also used datasets from GraphNovo (HeLa, Cerebellum, Plasma, A. thaliana, C. elegans, and E. coli). These datasets were used in the GraphNovo paper [1]. The HeLa and Cerebellum datasets were used for training, the Plasma dataset were used for validation, and the A. thaliana, C. elegans, and E. coli datasets were used for testing. For these datasets, SEQUEST through Proteome Discoverer 2.5 (Thermo Fisher Scientific) was run, with fixed modification of the carbamidomethylation of cysteine (C) and variable modifications of the oxidation of methionine (M).


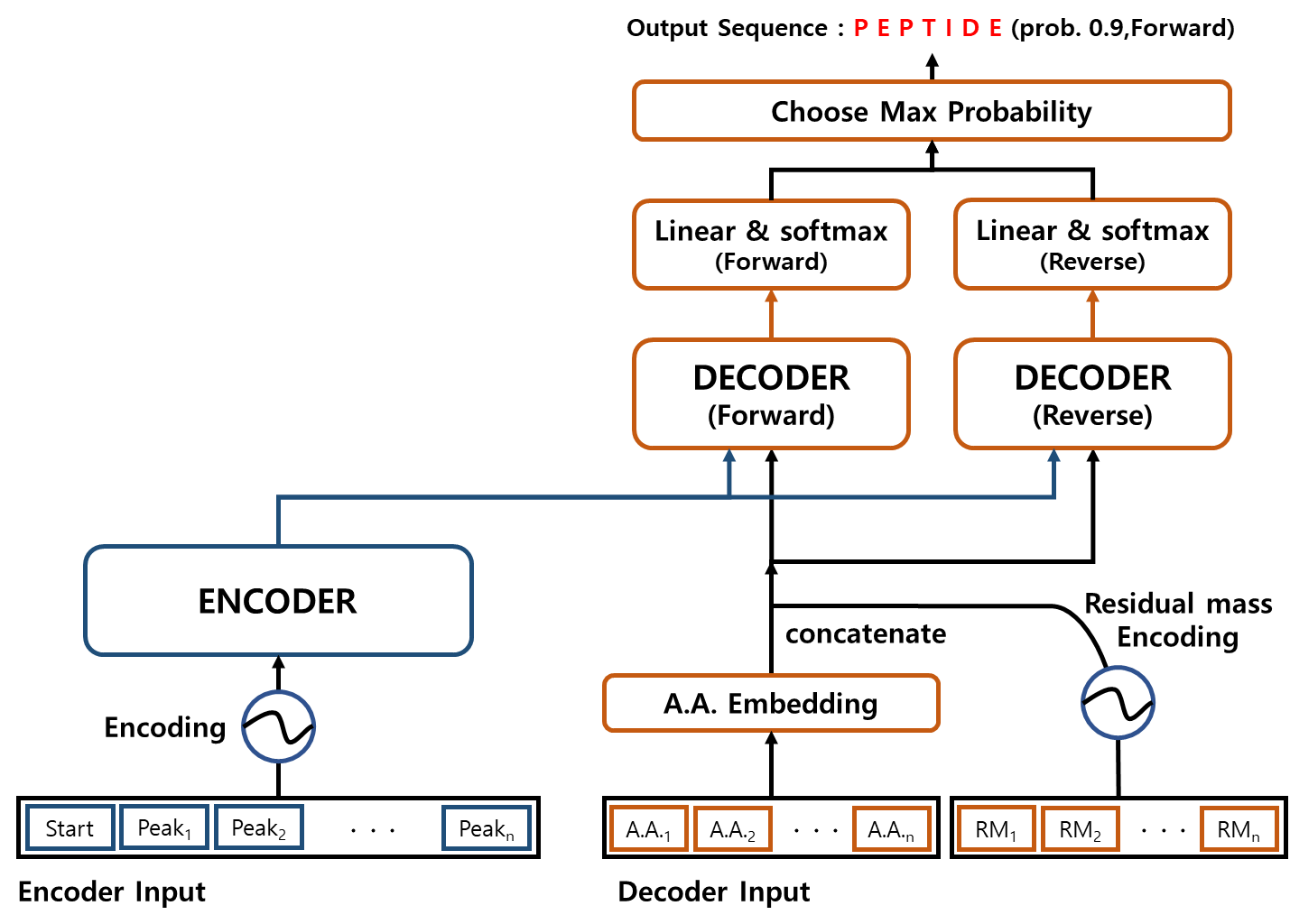


**Fig A in S1 Text |** Model architecture of NovoB

**Fig B in S1 Text | Comparison of DeepNovo, PointNovo, Casanovo, GraphNovo and NovoB (Bidirection).** These figures show the peptide-level and amino acid-level accuracy rates of five models on all three species.

**Table C in S1 Text |** **Comparison of the accuracy rates of NovoB, Casanovo, DeepNovo, and PointNovo.** The table presents the predictive accuracy rates at both the peptide level and amino acid level for the four models. The accuracy rates for all models were calculated with the coverage set to 1.

|  | **Peptide-level performance** | | | | **Amino-acid-level performance** | | | |
| --- | --- | --- | --- | --- | --- | --- | --- | --- |
| Species | DeepNovo | PointNovo | Casanovo | **NovoB** | DeepNovo | PointNovo | Casanovo | **NovoB** |
| Mouse | 0.286 | 0.355 | 0.443 | **0.465** | 0.623 | 0.626 | 0.562 | **0.735** |
| Human | 0.293 | 0.351 | 0.367 | **0.431** | 0.610 | 0.606 | 0.424 | **0.663** |
| Yeast | 0.462 | 0.534 | 0.561 | **0.661** | 0.750 | 0.779 | 0.591 | **0.825** |
| M mazei | 0.422 | 0.478 | 0.486 | **0.574** | 0.694 | 0.712 | 0.518 | **0.773** |
| Honeybee | 0.330 | 0.396 | 0.408 | **0.505** | 0.630 | 0.644 | 0.461 | **0.696** |
| Tomato | 0.454 | 0.513 | 0.460 | **0.588** | 0.731 | 0.733 | 0.471 | **0.775** |
| Rice bean | 0.436 | 0.511 | 0.437 | **0.661** | 0.679 | 0.730 | 0.442 | **0.814** |
| Bacillus | 0.449 | 0.518 | 0.540 | **0.634** | 0.742 | 0.768 | 0.573 | **0.811** |
| Clam bacteria | 0.253 | 0.298 | 0.371 | **0.433** | 0.602 | 0.589 | 0.405 | **0.681** |

**Table D in S1 Text |** **Summary of the learning times.** The table presents the number of batches, the average time (in seconds) for one epoch and 30 epochs, the average time (in hours) for 30 epochs, and the number of instances learned per second.

| Species | #Batch | Average time(s)  1 epoch | Average time(s)  30 epochs | Average time(h)  30 epochs | #learnings per seconds |
| --- | --- | --- | --- | --- | --- |
| Bacillus | 4,609 | 3,109.13 | 93,274 | 25.91 | 379.50 |
| Clam bacteria | 5,156 | 3,296.10 | 98,883 | 27.47 | 400.45 |
| Honeybee | 4,505 | 2,962.20 | 88,866 | 24.69 | 389.33 |
| Human | 5,214 | 3,323.03 | 99,691 | 27.69 | 401.68 |
| M mazei | 5,064 | 3,285.63 | 98,569 | 27.38 | 394.56 |
| Mouse | 5,551 | 3,442.40 | 103,272 | 28.69 | 412.81 |
| Rice bean | 5,554 | 3,455.83 | 103,675 | 28.80 | 411.43 |
| Tomato | 4,605 | 3,086.23 | 92,587 | 25.72 | 381.98 |
| Yeast | 5,304 | 3,365.13 | 100,954 | 28.04 | 403.50 |

[1] Mao, Z., et al. Mitigating the missing fragmentation problem in de novo peptide sequencing with a two stage graph-based deep learning model. Nature Machine Intelligence 2023.
